# Combined Effects of Ultrasound and Immobilization Protocol on Butyl Acetate Synthesis Catalyzed by CALB

**DOI:** 10.3390/molecules19079562

**Published:** 2014-07-07

**Authors:** Joana S. Alves, Cristina Garcia-Galan, Mirela F. Schein, Alexandre M. Silva, Oveimar Barbosa, Marco A. Z. Ayub, Roberto Fernandez-Lafuente, Rafael C. Rodrigues

**Affiliations:** 1 Biotechnology, Bioprocess and Biocatalysis Group, Institute of Food Science and Technology, Federal University of Rio Grande do Sul State, Av. Bento Gonçalves, 9500, P.O. Box 15090, Porto Alegre ZC 91501-970, RS, Brazil; E-Mails: joanasa.88@hotmail.com (J.S.A.); mi_schein@hotmail.com (M.F.S.); alexandremartins1990@gmail.com (A.M.S.); mazayub@ufrgs.br (M.A.Z.A.); 2 Department of Biocatalysis, ICP-CSIC. Campus UAM-CSIC. Cantoblanco, ZC 28049, Madrid, Spain; E-Mails: c.garcia@icp.csic.es (C.G.-G.); oveimar@gmail.com (O.B.); 3 Escuela de Química, Grupo de Investigación en Bioquímica y Microbiología (GIBIM), Edificio Camilo Torres 210, Universidad Industrial de Santander, Bucaramanga, 680001, Colombia

**Keywords:** esterification, lipase, ultrasound, enzyme reuse, enzyme stability, butyl acetate

## Abstract

It is well established that the performance of lipase B from *Candida* *antarctica* (CALB) as catalyst for esterification reactions may be improved by the use of ultrasound technology or by its immobilization on styrene-divinylbenzene beads (MCI-CALB). The present research evaluated the synthesis of butyl acetate using MCI-CALB under ultrasonic energy, comparing the results against those obtained using the commercial preparation, Novozym 435. The optimal conditions were determined using response surface methodology (RSM) evaluating the following parameters: reaction temperature, substrate molar ratio, amount of biocatalyst, and added water. The optimal conditions for butyl acetate synthesis catalyzed by MCI-CALB were: temperature, 48.8 °C; substrate molar ratio, 3.46:1 alcohol:acid; amount of biocatalyst, 7.5%; and added water 0.28%, both as substrate mass. Under these conditions, 90% of conversion was reached in 1.5 h. In terms of operational stability, MCI-CALB was reused in seven cycles while keeping 70% of its initial activity under ultrasonic energy. The support pore size and resistance are key points for the enzyme activity and stability under mechanical stirring. The use of ultrasound improved both activity and stability because of better homogeneity and reduced mechanical stress to the immobilized system.

## 1. Introduction

The use of lipases as catalysts for esterification reactions of short carboxylic acids has great interest because the esters thus obtained enzymatically may be labeled as “natural” [1]. These esters are used as flavor or fragrance ingredients by a variety of industries (food, pharmaceutical, cosmetics, *etc.*) [2]. Esterification is a simple reaction from a chemical point of view, consisting on the reaction between unmodified substrates, being the yields determined by the thermodynamic constant of the reaction.

Lipase-catalyzed esterifications have been performed in monophasic, low water activity systems, aiming at shifting the equilibrium towards the desired synthetic direction (water is a product of the esterification), usually using organic solvents [3,4], but also ionic liquids or supercritical fluids [5,6,7]. It has been shown that the water produced may be accumulated in the enzyme environment, producing its inhibition or even its inactivation. This problem may be solved by washing with solvents every few reaction cycles [8], or by using molecular sieves to remove the water from the medium [9]. Carboxylic acids and alcohols are also compounds capable of inactivating the enzyme. It has been suggested that the accumulation of all these substances is the main cause of observed lipase activity reduction during esterification reactions [8]. Therefore, an improved mixing of all reaction components might prevent the formation of an aqueous phase around the enzyme caused by a better dispersion of the carboxylic acids in the solvents, thus improving the enzyme performance. This improved mixing may be achieved by using ultrasound [10,11]. Ultrasound technology increases the interaction between phases by cavitation caused by the collapse of bubbles, and because of improved mixing, shearing, and mass transfer in aqueous solutions or suspensions [12,13]. Ultrasound technology has recently been used as an efficient way of mixing lipase-catalyzed reactions, such as transesterifications [14,15], or esterifications for the synthesis of isoascorbyl ester [16], sugar esters [17], and flavor esters [18,19]. However, few studies are found in the literature and scarce information is available on the mechanism(s) of ultrasound activation and its interaction with the enzymes and the immobilization supports.

The present work is part of a continued research project in our group in the field of flavor ester production, specifically of butyl acetate, an ester with apple notes, catalyzed by lipase B from *Candida antartica* (CALB) [8,19,20]. This lipase, in its free and immobilized forms, is the most used lipase, showing very good activity and stability properties [21]. In previous studies, Novozym 435 was used for butyl acetate synthesis obtaining high conversions (over 90%) in relatively short reaction times (2.5 h) [8]. Using ultrasonic energy, the process productivity was improved in the presence of higher amounts of substrate and the biocatalyst could be recycled for more reaction batches [19]. Additionally, CALB was immobilized via interfacial activation on very hydrophobic styrene-divinylbenzene beads (MCI-CALB) [22]. This preparation was also evaluated for the butyl acetate synthesis, where it displayed better performance than Novozym 435 under mechanical stirring [20]. 

In this context, the objective of this work was to analyze the performance of MCI-CALB as a catalyst for the esterification of acetic acid and butanol using ultrasound technology, comparing the effects with those previously found for Novozym 435 and with the same MCI-CALB, but using mechanical stirring. Therefore, reactions parameters were determined by an experimental design. Moreover, the effects of different carrier materials on the operational stability of the enzyme were investigated.

## 2. Results and Discussion

### 2.1. Experimental Design, Model Fitting and ANOVA

Initially, the four variables (temperature, substrate molar ratio, amount of biocatalyst, and added water) were evaluated measuring their effects on the initial reaction rate for the ultrasound-assisted butyl acetate synthesis catalyzed by MCI-CALB. The 27 experiments for the central composite design (CCD) with their results are shown on Table 1.

**Table 1:** Experimental design and results of CCD.

Treatment	X_1_	X_2_	X_3_	X_4_	Initial Reaction Rate (mmol·L^−1^h^−1^)
1	−1	−1	−1	−1	114.7
2	−1	−1	−1	1	92.9
3	−1	−1	1	−1	81.1
4	−1	−1	1	1	195.1
5	−1	1	−1	−1	82.2
6	−1	1	−1	1	98.2
7	−1	1	1	−1	223.7
8	−1	1	1	1	219.9
9	1	−1	−1	−1	202.1
10	1	−1	−1	1	146.2
11	1	−1	1	−1	264.0
12	1	−1	1	1	256.2
13	1	1	−1	−1	169.6
14	1	1	−1	1	99.9
15	1	1	1	−1	241.5
16	1	1	1	1	199.4
17	−2	0	0	0	140.4
18	2	0	0	0	143.9
19	0	−2	0	0	184.9
20	0	2	0	0	209.3
21	0	0	−2	0	44.5
22	0	0	2	0	273.9
23	0	0	0	−2	231.6
24	0	0	0	2	240.3
25	0	0	0	0	240.0
26	0	0	0	0	234.8
27	0	0	0	0	232.1

X_1_: temperature; X_2_: substrate molar ratio; X_3_: amount of biocatalyst; X_4_: added water.

The highest reaction rate (273.9 mmol·L^−1^h^−1^) was obtained in treatment 22 (45 °C, 3:1 alcohol:acid, amount of biocatalyst 10%, added water 0.5%), whereas the lowest activity was in treatment 21, with a reaction rate of 44.5 mmol·L^−1^h^−1^ (45 °C, 3:1 alcohol:acid, amount of biocatalyst 1%, added water 0.5%). In general, half of the treatments presented good reaction rates, over 200 mmol·L^−1^h^−1^, which represents almost 70% of conversion in 1 h. These were better results than those obtained using CALB for the butyl acetate synthesis in ionic liquids [23].

According to Fisher’s statistical test for analysis of variance (ANOVA), the model was statistically significant and adequate to represent the actual relationship between the responses and the variables, as suggested by the model F-value (6.51) and the very low p-value (*p* = 0.0012). The values of the determination coefficient, R^2^, and correlation coefficient, R, were, 0.88 and 0.94, respectively. This denotes a highly satisfactory representation of the process model and a good correlation between the experimental results and the theoretical values predicted by the model equation. The coefficients of variables were determined for the second-order polynomial model and the statistical significant (5%) are given below:

Y = 232.46 + 19.91X_1_ + 47.24X_3_ − 2.24X_4_ − 25.19X_1_^2^ − 11.45X_2_^2^ − 20.92X_3_^2^ − 18.63X_1_X_2_ − 17.49X_1_X_4_ + 12.13X_2_X_3_ − 8.00X_2_X_4_ − 11.98X_3_X_4_ (1)

where Y is the percentage conversion, and X_1_, X_2_, X_3_, and X_4_ are the coded values of temperature, substrate molar ratio, amount of biocatalyst and added water, respectively.

### 2.2. Effect of Process Parameters and Optimal Conditions

The obtained results can only describe the reaction within the range of variable values investigated in the experimental design. The linear effects of the four variables were: temperature (39.9), substrate molar ratio (2.5), amount of biocatalyst (94.5), and added water (−4.5). Only substrate molar ratio was not statistically significant at a 95% confidence level, in contrast with results seen when using Novozym 435 [19]. Reaction temperature and amount of biocatalyst presented the highest effects in the ultrasound-assisted butyl acetate synthesis catalyzed by MCI-CALB. The increase in both variables leads to an increase in the initial reaction rate. However, added water presented a negative effect on the initial reaction rate, which means that increasing the initial water content decreases the reaction rate. The tendency of the effect of these three variables was similar to that found using Novozym 435, although optimal values were slightly different. Thus, some differences could be found in this preliminary study comparing Novozym 435 and MCI-CALB [19].

**Figure 1:**
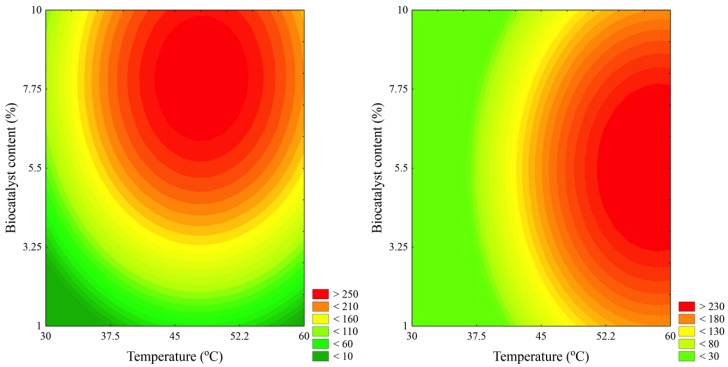
Contour plots for the ultrasound-assisted synthesis of butyl acetate catalyzed by MCI-CALB. (**a**) Condition I: Substrate molar ratio was fixed at level 0.46 (3.46:1 alcohol:acid) and added water was fixed at level −0.8 (0.28%); (**b**) Condition II: Substrate molar ratio was fixed at level −2 (1:1 alcohol:acid) and added water was fixed at level −2 (0%).

The optimal conditions for the ultrasound-assisted butyl acetate synthesis catalyzed by MCI-CALB were found using the software Statistica 7.0, and were: temperature, 48.8 °C; substrate molar ratio, 3.46:1 alcohol:acid; amount of biocatalyst, 7.5%; and added water 0.28%, both by substrate mass. This could be better observed through the contour plots in Figure 1a, where it is possible to obtain an initial reaction rate over 250 mmol·L^−1^h^−1^. These conditions were similar to those found when Novozym 435 was used as catalyst under ultrasound stirring [19], but it was quite different from the optimal conditions for MCI-CALB when the mechanical stirring was used [20]. However, using our previous experience, we fixed substrate molar ratio and water content at their lowest level (−2), since substrate molar ratio was not statistically significant and added water showed a negative effect, and a new curve was plotted. This curve was represented in Figure 1b and it is possible to observe a new optimal condition: temperature, 60 °C; substrate molar ratio, 1:1 alcohol:acid; amount of biocatalyst, 5%; and added water 0%, both by substrate mass. This optimal condition differs from the previous regarding the two most important variables, temperature and amount of biocatalyst. In the optimal condition I, a lower temperature was used at the expense of higher amount of biocatalyst, whereas at the optimal condition II (see Figure 1b), it was possible to use lower amounts of biocatalyst and increase the temperature.

**Figure 2:**
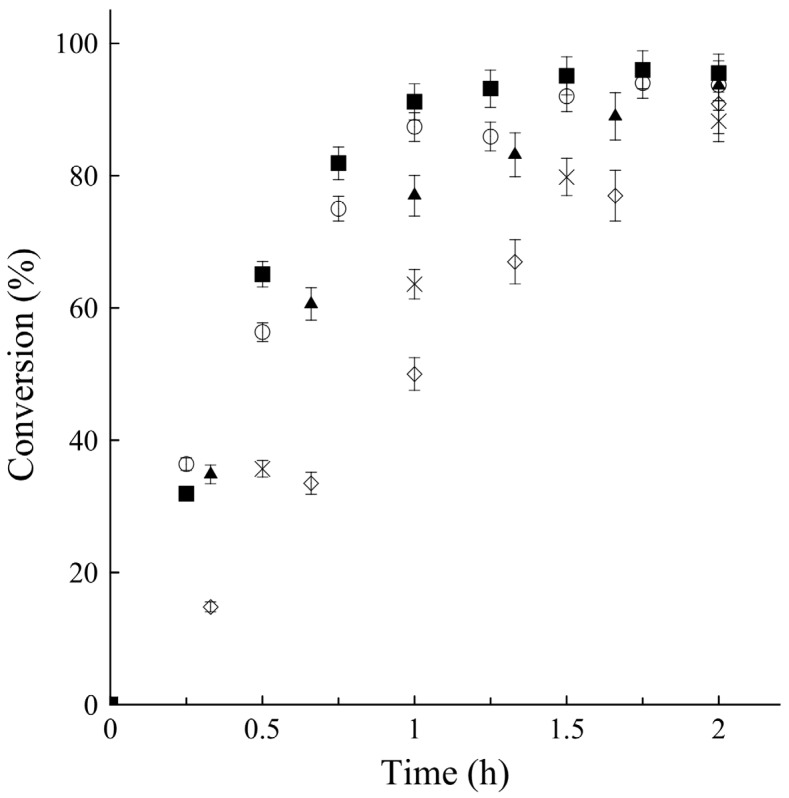
Time-course of butyl acetate synthesis at 0.3 M acetic acid: (■) MCI-CALB under optimal condition I; (○) MCI-CALB under Condition II; (◇) MCI-CALB under mechanical stirring conditions; (▲) Novozym 435 under ultrasound conditions; (×) Novozym 435 mechanical stirring conditions.

For model validation, experiments under both optimal conditions were performed, and the results of time courses for the ultrasound assisted butyl acetate catalyzed by MCI-CALB are shown in Figure 2, as well as the comparison with the previous results. The predicted values by the model were 276.2 and 248.4 mmol·L^−1^h^−1^, whereas the observed values were 278.7 and 256.5 mmol·L^−1^h^−1^ for conditions I and II, respectively, showing a good correlation of the experimental results with the statistical predicted by the model.

As it can be seen in Figure 2, the reaction courses were quite similar under both conditions, *i.e.*, it is possible to reduce the enzyme content while increasing the reaction temperature and reaching the same conversion. Higher than 90% of conversion was obtained after 1 h, which is much better than the results obtained using mechanical stirring (50% in 1 h, and over 90% in 2 h) [20], or when using Novozym 435 under ultrasound-assisted conditions (less than 80% in 1 h, and over 90% in 1.5 h) [19]. In conclusion, MCI-CALB used under ultrasonic energy was two times faster than when used under mechanical stirring [20] It was also 2.5 and 1.5 times more rapid than Novozym 435 under mechanical stirring and ultrasonic energy, respectively [8,19].

### 2.3. Enzyme Reuse

**Figure 3:**
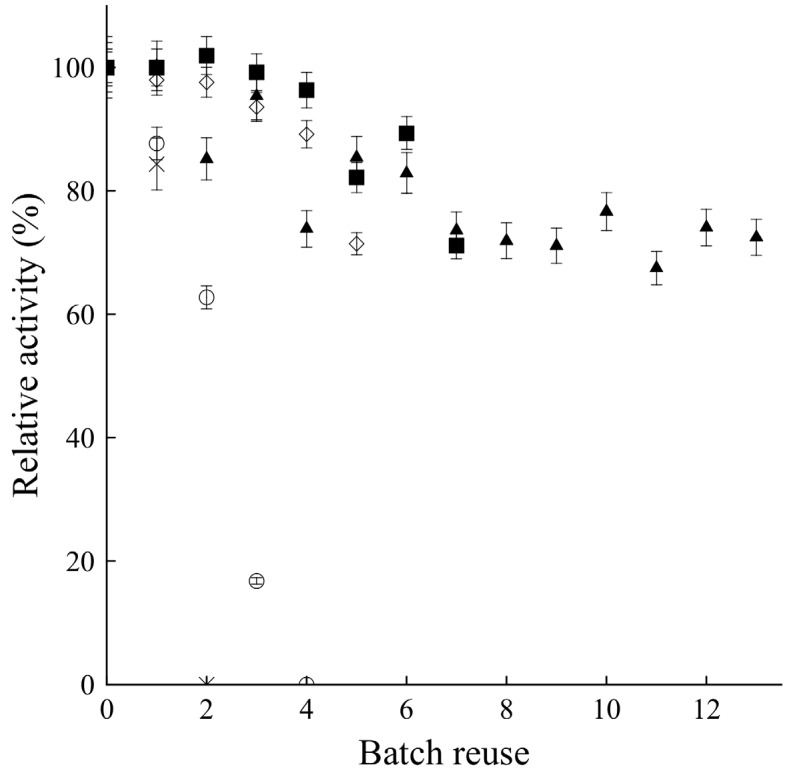
Operational stability of CALB during repeated batches on butyl acetate synthesis. (■) MCI-CALB under optimal condition I; (○)MCI-CALB under optimal Condition II; (◇) MCI-CALB under mechanical stirring conditions; (▲) Novozym 435 under ultrasound conditions; (×) Novozym 435 under mechanical stirring conditions.

Since the immobilized enzyme showed similar activities under both conditions, the other relevant parameter to be considered for the final choice between the use of higher temperature with lower amount of biocatalyst or the use of more biocatalyst and lower temperature, is the operational stability of the enzyme. The reuse of the biocatalyst under conditions I and II was tested, and the results of the repeated batches for ultrasound-assisted butyl acetate synthesis are presented in Figure 3. After four reuses under conditions II (lower amount of biocatalyst and higher temperature), the MCI-CALB was fully inactivated, whereas under condition I, the biocatalyst still kept over 70% of its initial activity after seven reuses. As expected, the effect of temperature improved enzyme activity, but simultaneously decreased enzyme stability. Although the reaction rates were similar, because the operational stability of the biocatalyst under condition I was higher, this was selected for the next experiments.

MCI-CALB showed slightly higher activity and stability under ultrasound stirring when compared to mechanical stirring, [20]. In relation to Novozym 435, one of the advantages of MCI-CALB under standard stirring was the increased operational stability of the enzyme in esterification reactions. However, under ultrasound stirring, the stability of Novozym 435 was strongly increased, whereas for MCI-CALB the improvement was small under condition I [19]. Thus, in the ultrasound-assisted reaction, the operational stability for MCI-CALB in the condition I was around half of the presented by the commercial preparation under its respective optimal conditions. The main advantage of the new preparation was the activity, which is 1.5-fold higher than Novozym 435.

### 2.4. Effect of Acid Concentration on Enzyme Activity

As demonstrated for Novozym 435, it is possible to use a higher acid concentration under ultrasonic energy when compared to mechanical stirring [19]. This fact might be explained by the better mixing of the acetic acid in the organic solvent caused by ultrasounds, which prevents the acid from becoming accumulated in the microenvironment of the enzyme, perhaps also avoiding the formation of a water phase in this microenvironment that could inactivate the enzyme [8].

**Figure 4:**
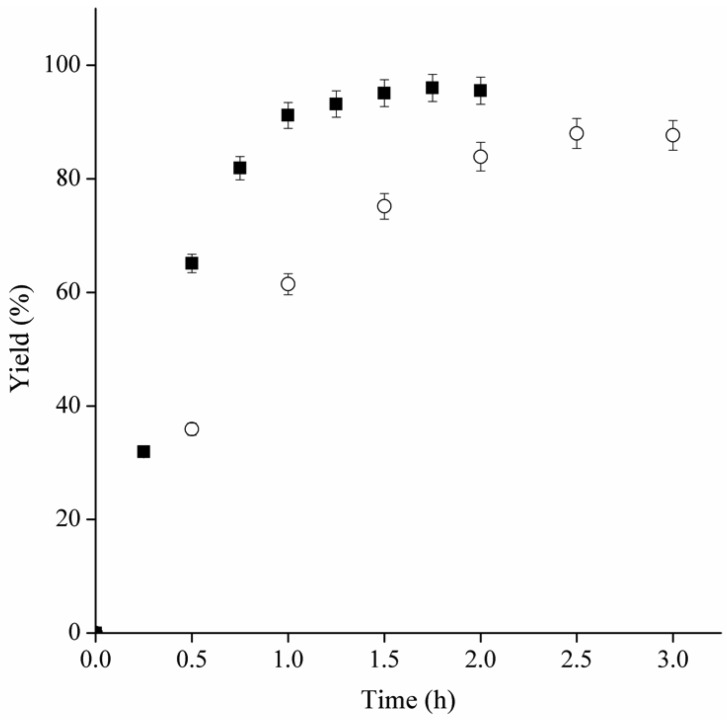
Time course of ultrasound-assisted butyl acetate synthesis catalyzed by MCI-CALB under condition I (temperature, 48.8 °C; substrate molar ratio, 3.46:1 alcohol:acid; amount of biocatalyst, 7.5%; and added water 0.28%) at (○) 2.0 M and (■) 0.3M acetic acid.

The time course of ultrasound-assisted esterifications with 0.3 M and 2.0 M acid concentrations are presented in Figure 4. Using 0.3 M, conversion reached to 97% after 1.5 h, whereas the conversion was 90% after 2.5 h when 2.0 M acetic acid was used. In terms of productivity, the ultrasound-assisted butyl acetate synthesis catalyzed by MCI-CALB using 2.0 M of acetic acid represents approximately 840 mmol·L^−1^h^−1^, which is six times higher than the productivity in the same reaction, but with mechanical stirring, (140 mmol·L^−1^h^−1^) [20]. Additionally considering the 1.5-fold enhancement in the operational stability caused by the ultrasound technology, the final improvement in the overall process productivity is almost 10 times higher.

### 2.5. Discussion

As part of a continued research effort studying esterification catalyzed by lipases, we improved the results reported in the literature by developing a fast and efficient procedure for the synthesis of butyl acetate catalyzed by Novozym 435 [8]. Furthermore, biocatalyst activities and operational stabilities were enhanced by the development of an immobilized system on styrene-divinylbenzene beads [20]. Novozym 435 was then used as a catalyst under the same reaction conditions, but using ultrasonic energy, allowing the use of seven times higher substrate concentration, thus obtaining improved activity and much higher stability of the biocatalyst [19]. Based on these facts, we raised the hypothesis that the use of MCI-CALB under ultrasonic energy could enhance enzyme activities and stabilities, when compared to previous researches [8,19,20]. However, there is a complexity in this apparently simple process that requires explanation. This complexity starts with the differences between the biocatalysts used. A summary of the properties of both biocatalysts is presented in Table 2.

Novozym 435 is the commercial preparation of Novozymes for immobilized CALB. The support used is Lewatit VP OC 1600, which is a macroporous, DVB-crosslinked polymer in spherical bead form, based on methacrylic esters, according to the supplier. Lewatit support has a particle size of 300–800 μm, which is adequate for filtration recovery for multiple reuses [24]. MCI-CALB was prepared by the immobilization through interfacial activation of CALB on the MCI GEL CHP20P [22]. This support has a styrene-divinylbenzene matrix and it has a smaller particle diameter than Lewatit VP OC 1600, 75–150 μm [24]. The structural differences of both supports, in terms of matrix and particle size, could affect the mechanical resistance, thus the operational stabilities. Comparing both preparations under mechanical stirring, MCI-CALB presented higher activity and operational stability than Novozym 435. The better performance of MCI-CALB can be explained by the following facts. MCI GEL CHP20P has higher pore diameter than Lewatit VP OC 1600. The mean pore size of MCI GEL CHP20P is around 400–600 Å [22], whereas for Lewatit VP OC 1600 it is around 150–300 Å [24,25]. The higher pore size eases the substrates/products mass transfer through the support and may explain the higher activity of MCI-CALB. Additionally, Lewatit VP OC 1600 is by far more hydrophilic than styrene-divinylbenzene beads of MCI GEL CHP20P. This might facilitate the accumulation of water, acid, and alcohol in the enzyme environment, reducing enzyme operational stability. This accumulation of substrates and products in the enzyme environment may be strongly reduced using ultrasound stirring technology. Moreover, Lewatit VP OC 1600 has shown to have a relatively low mechanical resistance [24]. In stirred-reactor systems, fragmentation of the carrier can be observed, resulting in decreased enzymatic activity by failure in enzyme recovery [26]. Thus, the lower operational stability of Novozym 435 under mechanical stirring could also be attributed to fragmentation, whereas the ultrasound stirring does not promote such negative effect on the bead integrity.

**Table 2:** Summary of the properties of the biocatalysts.

	MCI-CALB	Novozym 435
Support	MCI GEL CHP20P	Lewatit VP OC 1600
Matrix	Styrene-divinylbenzene	DVB-crosslinked polymer based on methacrylic esters
Particle size	75–150 μm	300–800 μm
Pore size	400–600 Å	150–300 Å
Mass transfer	High	Low
Hydrophilicity	Low	High

Nevertheless, it was observed that MCI-CALB did not present any significant advantage over Novozym 435 for the esterification reaction under ultrasound energy. There are also some possible reasons for this. Although MCI-CALB has higher pore size than Novozym 435, and this was an advantage under mechanical stirring, the use of ultrasound allowed better mixing in the reaction mixture, reducing the mass transfer limitations. It was reported by other authors that the use of mechanical stirring for enzymatic esterification using immobilized enzymes generally has a limitation in internal mass transfer that might reduce the rate of ester formation [27,28]. Moreover, the use of ultrasounds allowed the increase in the concentration of substrate that could be used in the reaction. The maximum concentration was improved seven times for Novozym 435 and four times for MCI-CALB. Besides the reduction of the mass transfer limitations, the more homogeneous mixing obtained under ultrasound avoids substrate/product/water gradients inside the porous matrix of the support. Avoiding the formation of water layer surrounding the enzyme, the acid will not be concentrated in the enzyme environment, which could lead to a lower pH, thus decreasing the enzyme stability. In addition, the use of ultrasounds reduced the mechanical stress caused by mechanical stirring, thus Novozym 435 presented better operational stability than MCI-CALB under ultrasound. The higher particle size of Lewatit VP OC 1600 as compared to MCI GEL CHP20P allowed an easier separation and recovery of the biocatalyst, which is important in the reusability.

Nevertheless, results strongly support that ultrasound energy improved the activity and the operational stability for both biocatalysts. In the present research, MCI-CALB preparation was quite stable under operational conditions, and it could be reused several cycles without any washings, even under standard stirring techniques. Ultrasound technology improved this operational stability 1.5 times and the activity was twice as high as compared to mechanical stirring. Novozym 435 also improved the same parameters under ultrasonic technology. The effect of this technology was even more significant in the operational stability of Novozym 435, as compared to MCI-CALB, making unnecessary the washings between reaction cycles, whereas it was almost fully inactivated under conventional stirring in only 2 to 3 cycles [8], for the reasons discussed above. Optimal conditions for Novozym 435 under ultrasound, as determined by RSM, were similar to those determined in this work for MCI-CALB: temperature of 46 °C; substrate molar ratio of 3.6:1 butanol:acetic acid; amount of biocatalyst of 7%; added water of 0.25%. Using 2 M acetic acid, 82% of conversion was reached using Novozym 435 after 2.5 h, whereas conversion of 90% was obtained using MCI-CALB in the same time, even when the amount of protein per mass of dried biocatalyst was about two times higher for Novozym 435 [20]. Therefore, even under ultrasound stirring that greatly improved the Novozym 435 performance, MCI-CALB remained with a better productivity per cycle than Novozym 435 per mass unit, and much higher comparing the productivity per enzyme molecule.

Another interesting characteristic associated to ultrasound technology is that it is considered a green technology because of its high efficiency, low instrumental requirements, and significant reduction of the processing time when compared to other techniques [29]. Moreover, is an excellent tool for improvement of chemical, physical and biological processes [12,28,30,31,32]. Ultrasound has been used in many applications, such as homogenizing, disintegration, sonochemistry, degassing and cleaning, extraction, emulsification, and chemical synthesis [33]. The mechanism of ultrasound is based on the high-energy waves that create cavitation in the liquid solution. The subsequent collapses of the cavitation bubbles release the energy. This mechanism is one of the factors that contribute to the acceleration of the chemical and/or enzymatic reaction in the solution [34].

## 3. Experimental Section

### 3.1. Materials

Lipase B from *Candida antarctica* was kindly donated by Novozymes (Madrid, Spain). The styrene–divinylbenzene MCI GEL CHP20P porous support, substrates, solvents, and other chemicals were purchased from Sigma-Aldrich (Sigma, St. Louis, MO, USA) and were of analytical grade. Ultrasonic bath (Unique, model USC 2880A, 40 kHz, 220 W, Indaiatuba, Brazil) with temperature control was used in all experiments.

### 3.2. Enzyme Immobilization

The immobilization of CALB on the styrene-divinylbenzene support was carried out following a previously described protocol [22]. The immobilized preparations presented 120 mg of protein per g of wet support.

### 3.3. Esterification Reaction

The substrates *n*-butanol and acetic acid (0.3 M) [8] were dissolved in *n*-hexane at different molar ratios in 50 mL Erlenmeyer flasks (working volume of 10 mL), followed by the addition of various amounts of water and enzyme. The reaction was carried out in an ultrasonic bath, at various temperatures, according to the experimental design (Table 1).

The progress of the esterification was monitored determining the residual acid content by titration of 0.5 mL of sample with NaOH (0.005 M) until pH 7, using ethanol as quenching agent. The amount of ester was calculated as being equivalent to the consumed acid. A calibration curve was constructed to ensure the reliability of this acid determination using laboratory-made mixtures of acetic acid, *n*-butanol, and commercial butyl acetate in *n*-hexane. In some points, the accuracy of this method was also tested by the determination of ester concentration on gas chromatograph (Shimadzu, Tokyo, Japan, GC-2010 Plus), equipped with a flame ionization detector (FID) and an AT.FFAP column (30 m × 0.32 mm × 0.25 μm). The carrier gas was nitrogen. The temperatures of the injector and detector were both set to 250 °C, and the split ratio was 1:10. The oven temperature program was: start at 60 °C, 10 °C min^−1^ to 90 °C, 30 °C min^−1^ to 240 °C, and then held at 240 °C for 2.5 min.

### 3.4. Experimental Design

In order to obtain the optimal conditions for the esterification a central composite design (CCD) with four variables was performed. The four variables, each with five levels, are presented in Table 3 with their coded and uncoded values. The CCD, with 28 experiments, was composed of 16 factorial points, eight axial points (two axial points on the axis of design variable), and four replications at the central point. In each case, the initial reaction rate for esterification was calculated.

**Table 3:** Process variables and their levels used in CCD.

Variables	Name	Coded Levels				
		−2	−1	0	1	2
X_1_	Temperature (°C)	30	37.5	45	52.5	60
X_2_	Substrate Molar Ratio ^a^	1:1	2:1	3:1	4:1	5:1
X_3_	Amount of biocatalyst ^b^	1	2.5	5	7.5	10
X_4_	Added Water ^b^	0	0.25	0.5	0.75	1

^a^ (butanol:acetic acid); ^b^ (% by mass of substrate).

The second-order polynomial equation for the variables was as follows:

*Y* = *β*_0_ + ∑*β_i_X_i_* + ∑*β_ij_X_i_X_j_* + ∑*β_ii_X_i_*^2^ (2)

where Y is the response variable, β_0_ the constant, β_i_, β_ii_, β_ij_ were the coefficients for the linear, quadratic, and for the interaction effects, respectively, and X_i_ and X_j_ the coded level of variables x_i_ and x_j_. The above quadratic equation was used to plot surfaces for all variables.

### 3.5. Enzyme Reuse

After the esterification reaction, the immobilized enzyme was separated from the reaction medium by vacuum filtration using a sintered glass funnel, and placed in a fresh reaction batch.

### 3.6. Statistical Analysis

The experimental design and analysis of results were carried out using Statistica 12.0 (Statsoft, Tulsa, OK, USA). The statistical analysis of the model was performed as analysis of variance (ANOVA). The significance of the regression coefficients and the associated probabilities, p(t), were determined by Student’s t-test; the second order model equation significance was determined by Fisher’s F-test. The variance explained by the model is given by the multiple determination coefficients, R^2^. For each variable, the quadratic models were represented as contour plots (2D).

## 4. Conclusions

This work has demonstrated the improvement of the synthesis of butyl acetate catalyzed by MCI-CALB when using the more efficient homogenizing technology of ultrasound energy. The optimal conditions for butyl acetate synthesis by MCI-CALB were: temperature, 48.8 °C; substrate molar ratio, 3.46:1 alcohol:acid; amount of biocatalyst, 7.5%; and added water 0.28%, both by substrate mass. Under these conditions, 90% of conversion was reached in 1.5 h of reaction. The use of the ultrasound increased the process productivity by six times, allowing the use of higher (up to four-fold) acid concentrations, while reducing the reaction time compared to the classical mechanical stirring. Additionally, concerning the effects of the stirring system and the differences of supports, it was possible to conclude that:

- A higher pore size is better for substrate/products diffusion when using mechanical stirring;
- The pore size was not so significant on the diffusion rates when using ultrasound because the stirring was more vigorous and homogeneous;
- The lower mechanical stress caused by ultrasound improves operational stability, in special for more sensitive supports.
